# BAOXIN Granules Protected Mouse Model With Elevated Afterload From Cardiac Hypertrophy by Suppressing Both Inflammatory Reaction and Collagen Deposition

**DOI:** 10.3389/fphys.2019.00820

**Published:** 2019-07-05

**Authors:** Xu Qiu, Ji Ma, Yujing Shi, Dong Zhang, Defeng Li, Zhao Dong, Xiao Lin, Haozhe Shi, Guining Jiang, Yuhui Wang, George Liu

**Affiliations:** ^1^Key Laboratory of Molecular Cardiovascular Science Ministry of Education, Institute of Cardiovascular Science, Health Science Center, Peking University, Beijing, China; ^2^Jishantang Clinic of Traditional Chinese Medicine, Yinchuan, China; ^3^Institute of Chinese Materia Medica, China Academy of Chinese Medical Sciences, Beijing, China; ^4^Clinical Measurement, Cardiology Department, Westmead Hospital, Sydney, NSW, Australia

**Keywords:** BAOXIN Granules, cardiac hypertrophy, transverse aortic constriction, inflammation, fibrosis

## Abstract

BAOXIN Pill was reported to be effective clinically for chronic heart failure based on the principles of traditional Chinese medicine (TCM), invigorating *qi* and activating *blood*. The present study evaluated preclinically the effects of the improved dosage form, BAOXIN Granules, on cardiac hypertrophy. Transverse aortic constriction (TAC) was performed in mice to model cardiac hypertrophy by aortic stenosis for 4 weeks. The sham and TAC group were intragastrically administrated with saline as the controls. Two treatment groups were administrated orally with 10 mg/kg⋅d Enalapril (positive control) or 0.77 g/kg⋅d BAOXIN Granules for 4 weeks respectively. The effects were evaluated by echocardiography, morphology, and biological markers for cardiac function. The specific genes involved in inflammation and fibrosis were also examined for their expressions to investigate the pathways involved in early heart failure. Just as Enalapril, BAOXIN Granules administration markedly attenuated left ventricular hypertrophy and improved heart function as evidenced by echo cardiography, morphology. Accordingly, the biomarkers of the early stage heart failure, ANP, BNP and β-MHC, were decreased in the two treatment groups. We also found that mRNA expressions of some inflammatory factors and fibrosis associated genes were down-regulated in the tissue of heart after treatment. BAOXIN Granules may protect the heart from myocardial hypertrophy caused by increasing left ventricular afterload. It can suppress both inflammatory reaction and collagen deposition during pressure overload. BAOXIN Granules is advised to be tested in clinical trials for heart failure in the future.

## Introduction

Cardiac hypertrophy has been demonstrated to be an independent risk factor for morbidity and mortality of cardiovascular diseases (Levy et al., 1990; Reichek et al., 2009; Zile et al., 2014). Myocardial hypertrophy, with main feature of left ventricular mass and cardiomyocytes volume increases, is a compensatory reaction against various physiological and pathological process, such as hypertension, ischemic heart disease, and genetic cardiac defects (Komuro, 2001). Persistent and prolonged hypertrophic status is highly associated with arrhythmia and heart failure (Kim et al., 2004; Yang et al., 2013). Preventing the progress and even reversing the already presented cardiac hypertrophy is of great importance. In this status, heart undergoes a series of pathological processes such as energy metabolism, remodeling, and inflammation although detailed mechanisms remain to be further investigated (Allard et al., 1994; Reichek et al., 2009). Unfortunately, there is still lack of effective anti-hypertrophy intervention with little side effect.

Traditional Chinese medicine (TCM) has accumulated a wealth of clinical experience in treatment of heart failure, especially in alleviating symptoms and improving life quality (Hao et al., 2017). According to TCM opinion, heart failure is caused by deficiency of heart *qi* and stasis of *blood* (Wen-Ting et al., 2012; Zhu et al., 2016). BAOXIN Pills, a formulation developed according to this theory, has been reported clinically for heart failure treatment in Beijing Chaoyang TCM Hospital (Pan and Gao, 1998). Invigorating heart *qi* and activating *blood*, the cardiac function was marked improved in those patients with chronic heart failure after treatment. The primary bioactive ingredients in each crude drug of BAOXIN Pills have been reported to affect the pathological processes involved in heart failure (briefly listed as Supplementary Table 1). For example, Astragaloside, a main active ingredient extracted from Astragalus, could exert its anti-hypertrophic effect through attenuating inflammatory cytokines (Komuro, 2001) and regulating energy biosynthesis (Zhang et al., 2015); Tanshinone is obtained from the root of Salvia miltiorrhiza and has multiple pharmacological activities, such as preventing cardiac fibrosis (Maki et al., 2002), modulating collagen metabolism (Ling et al., 2009), and reducing oxidative stress (Hu et al., 2015; Jeong et al., 2016); Angelica extract can improve cell apoptosis induced by Angiotensin II (Huang et al., 2014a); Poria cocos is used for its diuretic effect in TCM (Feng et al., 2013). Because of the complicated components in the preparations of TCM, the protective effects on the heart must due to pharmacological actions on multiple targets.

BAOXIN Pills is prepared by traditional simple process with pulverizing crude herbs, and there has no pre-clinical study for pharmacological mechanism in animal disease model yet. BAOXIN Granules, a new dosage form developed by China Academy of Chinese Medical Sciences through refined purification, is more convenient for clinical application. Here, we investigated the effects of this new preparation in a transverse aortic constriction (TAC) mouse model.

Transverse aortic constriction, by surgical ligation of the transverse aorta, can cause compensated cardiac hypertrophy and remodeling (Mohammed et al., 2012). This model can mimic human cardiac hypertrophy and even heart failure (Hasenfuss, 1998) to assess drug action response to pressure overload and associated mechanisms (Balasubramanian et al., 2015; Wu et al., 2015, 2016). Therefore, the present study was carried out by this model to affirm protective effects against cardiac hypertrophy of BAOXIN Granules. We also investigated its influence on expressions of inflammation and fibrosis related genes to understand the underlying mechanisms.

## Materials and Methods

### Animals

Specific pathogen-free female ICR mice 8 weeks of age were used in this study. They were purchased from Beijing Vital River Laboratory Animal Technology, Co., Ltd., (Vital River). The mice were housed in IVC under controlled conditions in the facility of Department of Laboratory Animal Science in Peking University Health Science Center. The facility is managed by Vital River. The mice were fed with chow diet *ad libitum* with the light–dark periods of 12 h. The experimental procedures were conducted under the guide of Care and Use of Laboratory Animals published by the National Institutes of Health (NIH), United States. Animal experiments were approved by the Animal Care Committee of Peking University Health Science Center (No. LA2015012) and the efforts were made to minimize animal suffering and reduce the number used.

**FIGURE 1:**
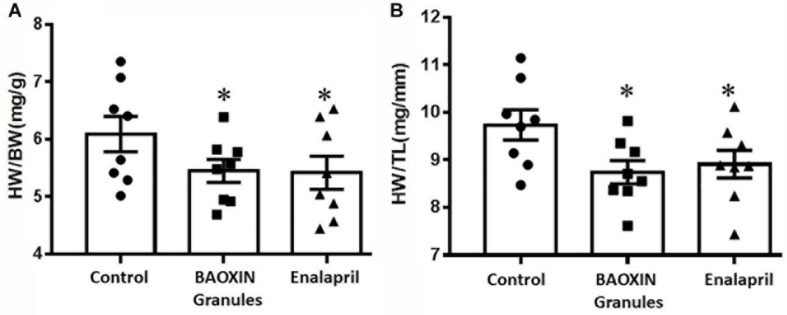
Effects of BAOXIN Granules and enalapril on mice heart weight (HW) which were subjected to TAC surgery. **(A)** Ratio of heart weight to body weight (HW/BW); **(B)** Ratio of heart weight to tibia length (HW/TL). The data were showed as mean and standard error of the mean (mean ± SEM). ^*^p < 0.05 compared to Control group, *n* = 8.

After 4 weeks for recovery from TAC operation, the mice were randomly divided into four groups (*n* = 8) for gastric lavage with saline (negative control, indicated as Control), Enalapril (positive control) at 10 mg/kg⋅d, BAOXIN Granules at 0.77 g/kg⋅d, and sham-operated group with saline (indicated as Sham), respectively. The dosage of BAOXIN Granules used in this study was calculated through the surface area formula, which was equally effective to recommended human dosage (Pan and Gao, 1998). Enalapril (batch number 014120503) was manufactured by CSPC Ouyi Pharmaceutical Co., Ltd. BAOXIN Granules was prepared by Institute of Chinese Materia Medica, China Academy of Chinese Medical Sciences as described in the Supplementary Materials. The orthogonal experimental results of process were briefly showed as Figure 1 and Supplementary Tables 2, 3. Both drugs were dissolved in saline. Adverse events during the experimental period were assessed by monitoring body weight (BW) and daily activities. The cardiac function was tested by echocardiography after 4 weeks of administration, and then the mice were sacrificed under 5% chloral hydrate (350 mg/kg) anesthesia.

### Generation of Animal Model

The operation of TAC was performed as described previously (deAlmeida et al., 2010). Briefly, the mice were anesthetized by intraperitoneal injection of 5% chloral hydrate (350 mg/kg) to relieve suffering, and then were connected to a rodent ventilator cycling (150 breaths/minute and 0.2 ml tidal volume) by endotracheal intubation. A silk suture was placed under the isolated aorta between the origin of the right innominate and the left common carotid arteries after opened the chest. The ascending aorta was snugly tied around the aorta with a 26G blunted needle and then removing the needle. After the suture, the mice were injected intraperitoneally with Benzylpenicillin (manufactured by Huabei Pharmaceutical, Co., Ltd.) to prevent infection.

### Echocardiographic Analysis

Mice were anesthetized through inhaling 1% isofluorane/oxygen for echocardiogram using Vevo 770 ultrasound system (FujiFilm Visual Sonics, Inc.) with a 17.5 MHz linear array transducer. The left ventricular (LV) cavity diameter, posterior and anterior wall thickness were measured (shown as Supplementary Table 5). The cardiac function was evaluated by the following formulae:

LV⁢fractional⁢shortening⁢(FS%)=100×[(LVIDd-LVIDs)/LVIDd](LVIDd⁢is⁢the⁢abbreviation⁢for⁢LV⁢internal⁢diameter⁢in⁢diastole;LVIDs⁢is⁢the⁢abbreviation⁢for⁢LV⁢internal⁢diameter⁢in⁢systole);LVmass=1.04×(LVIDd+LVPWd+LVAWd)-3LVIDd;3LVejectionfraction(EF%)=[(LVEDD-3LVESD)3/LVEDD]3×100;

(LVEDD is the abbreviation for LV end-of-diastolic diameter; LVESD is the abbreviation for LV end-of-systolic diameter).

### Histological Examination

The mice were perfused with cardioplegic solution containing 120 mEq/L of KCl and 100 mEq/L of NaCl as reported previously (Rabkin et al., 1998), and then 10% neutral-buffered formalin for hearts collected. After fixed in 4% formaldehyde heart tissues were dealt with through dehydration, clearing as well as wax immersion and then embedded in paraffin. The blocks were then sectioned (3 μm) and stained with hematoxylin and eosin (HE) and Sirius Red for morphological evaluation under a light microscope (Leica, Germany) and photographed in different magnification. The sections were also stained with fluorescein isothiocyanate-labeled wheat germ agglutinin (Sigma) for 1 h at room temperature as reported (Baudouy et al., 2017). We measured 50 cells/section at the level of the nucleus and 3 sections for one mice hearts. Cardiomyocyte cross-section width and fibrosis area were analyzed by Image J 1.46R.

### RNA Isolation and Quantitative PCR Analysis

Total RNA was extracted from heart tissues by Trizol reagent (Invitrogen, United States) and subjected to reverse transcription using an RT kit (Invitrogen, United States). The procedure of real-time PCR was followed the manufacturer’s instructions of SYBR Green (Invitrogen, Carlsbad, CA, United States). Relative quantitation of gene expression used the comparative CT method normalized to GAPDH (Hu et al., 2015) and the data are showed as the multiples of the sham. Specific primers for mice of atrial natriuretic peptide (ANP), brain natriuretic peptide (BNP), β-MHC, IL-1β, IL-6, TNF-a, Collagen I, Collagen III and GAPDH were list in Supplementary Table 4.

### Enzyme-Linked Immunosorbent Assay

The concentrations of cytokines IL-1β in the blood from all groups were measured using a commercially available ELISA kit (mouse IL-1β ELISA kit, R&D Systems, Inc., United States). Briefly, blood samples were centrifuged at 3000 rpm for 15 min at 4°C, and then ELISA assay was performed according to the manufacturer’s instructions.

### Statistical Analysis

All the numerical data were showed as mean and standard error of the mean (mean ± SEM). Statistical significance analysis was performed using GraphPad Prism 5.01 with one-way analysis of variance. It was considered as a statistically significant difference compared with control group when *p* < 0.05.

## Results

### BAOXIN Granules Treatment Attenuated Pathological Heart Hypertrophy

After 4 weeks of BAOXIN Granules treatment, no significant difference in BW was observed in four groups and the heart weight (HW) to BW ratio increased significantly in Control group compared to Sham Group (Supplementary Figure 2). HW to BW ratio (Figure 1A) and HW to tibia length (TL) ratio (Figure 1B) were decreased after BAOXIN Granules treatment, suggesting that its effect of prevention from heart hypertrophy caused by over-afterload in mice TAC model.

### BAOXIN Granules Treatment Improved Cardiac Function

**FIGURE 2:**
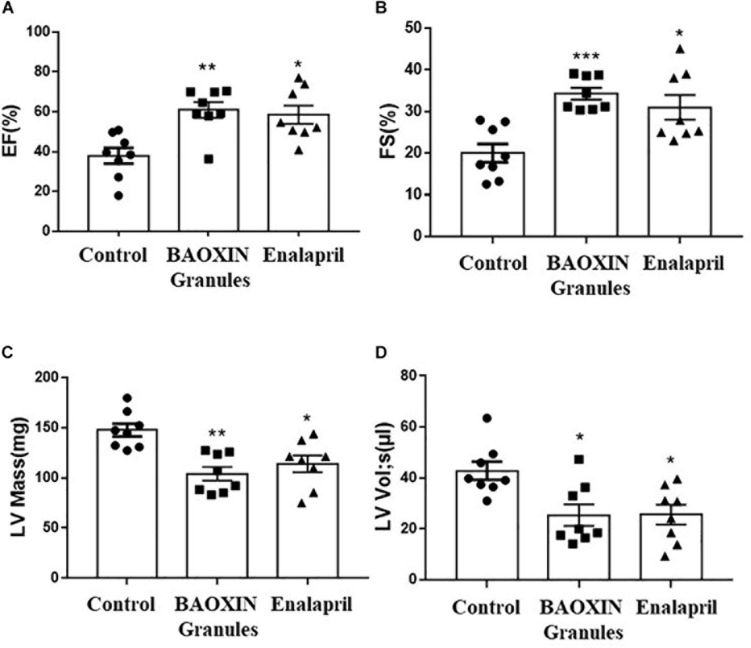
Echocardiographic analysis of cardiac function after 4 weeks treatments. LV systolic function was reflected by quantification of EF **(A)**, FS **(B)**, LV Mass **(C)**, LV Vol;s **(D)**. The data were showed as mean and standard error of the mean (mean ± SEM). ^*^p < 0.05 or ^∗∗^*p* < 0.01 compared with Control group, *n* = 8.

Eight weeks after the TAC surgery, we used two-dimensional (2D) guided M-mode echocardiography to assess the cardiac function of each group. We measured left ventricle (LV) anterior and posterior wall at diastole (LVAWd, LVPWd) (showed as Supplementary Tables 5, 6), which can reflect the structure of the LV, and calculated FS%, EF% and LV Mass as described above in methods. Compared with the control group, LV systolic function, as reflected by EF% (Figure 2A), FS% (Figure 2B), LV Mass (Figure 2C), and LV end-systolic volume (Figure 2D) was significantly increased in the BAOXIN Granules treated mice as well as Enalapril after TAC surgery. Therefore, both BAOXIN Granules and Enalapril significantly attenuated the decline of cardiac function induced by TAC surgery.

### BAOXIN Granules Reduced Cardiac Hypertrophy

**FIGURE 3:**
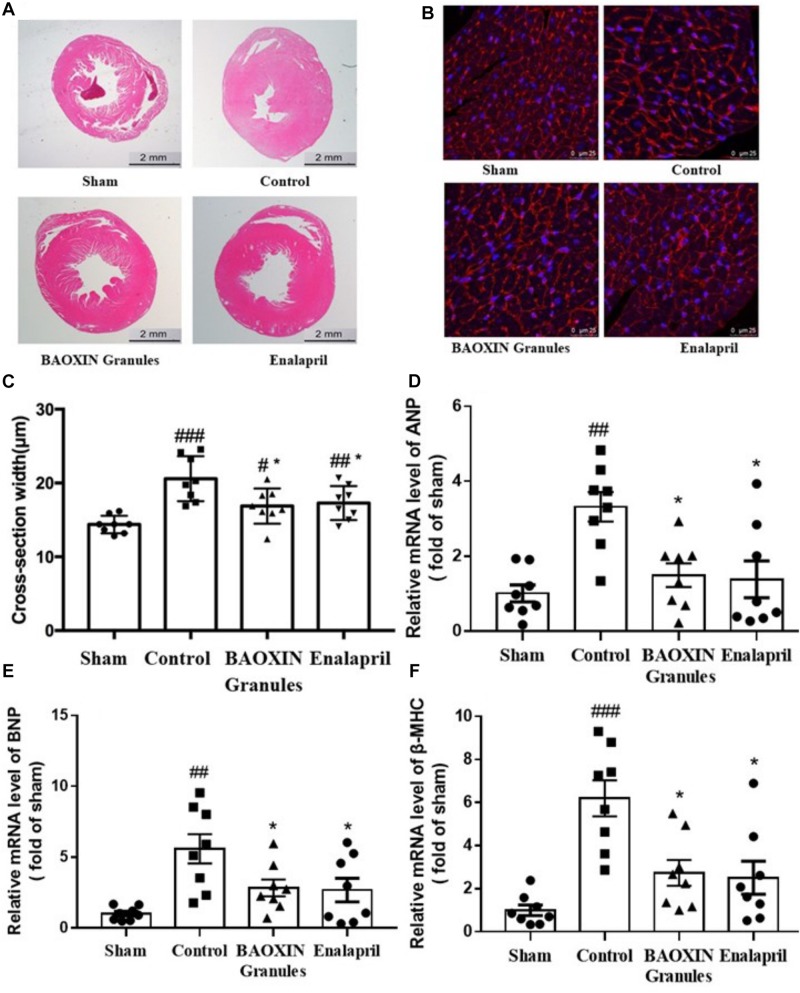
Morphological analysis of cardiac hypertrophy and the changes of some biomarkers. Representative photographs with left ventricular tissue sections in four groups by low (**A**, H.E. staining) and high (**B**, WGA staining) magnification. The cross-section width **(C)** was measured with the photos in high magnification (WGA staining) by the software ImageJ. Total 50 myocardial cells/section and three sections were measured in one sample. Hypertrophy biomarkers of ANP **(D)**, BNP **(E)**, and β-MHC **(F)** in heart tissues were evaluated by their mRNA levels. The numerical data were showed as mean and standard error of the mean (mean ± SEM). ${}^{\#}$*p* < 0.05, ${}^{\#\,\#}$*p* < 0.01, or ${}^{\#\,\#\,\#}$*p* < 0.001 compared with the Sham group; ^*^p < 0.05, ^∗∗^*p* < 0.01, or ^∗∗∗^*p* < 0.001 compared with the Control group, *n* = 8.

According to morphological analysis, LV hypertrophy became significantly more severe in TAC mice compared to sham group and was ameliorated after BAOXIN Granules treatment. As shown in Figure 3A, enlarged heart and thickened ventricular wall was obviously observed in TAC model, and BAOXIN Granules or Enalapril treatment attenuated cardiac hypertrophy compared with control group. The LV posterior wall thickness (LVPW) in systole and diastole (LVPWs and LVPWd) analyzed by echocardiography also showed the same tendency (Supplementary Table 5). The ventricular myocytes sizes also significantly increased in TAC groups as Figures 3B,C. At the same time, the mRNA expression of ANP, BNP, and β-MHC in heart (Figures 3D–F), molecular marker of cardiac hypertrophy, all markedly increased after TAC induced. However, BAOXIN Granules or Enalapril inhibited the ventricular myocytes sizes and downregulated the expressions of the molecular marker of cardiac hypertrophy.

### BAOXIN Granules Attenuated Inflammatory Response Induced by TAC

**FIGURE 4:**
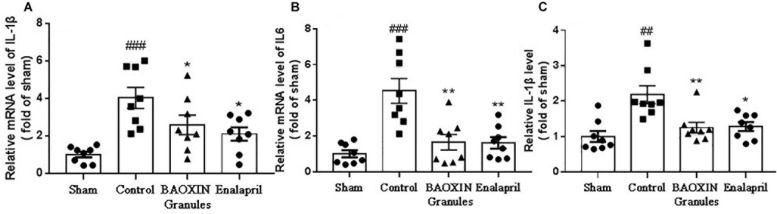
The detection of inflammatory factors in heart tissue and blood. The mRNA levels were tested by real time PCR in heart tissues for inflammatory factors of IL-1β **(A)** and IL-6 **(B)**. The IL-1β in blood **(C)** was assayed by ELISA. The data were showed as mean and standard error of the mean (mean ± SEM). ${}^{\#\,\#}$*p* < 0.01 or ${}^{\#\,\#\,\#}$*p* < 0.001 compared with the Sham group. ^*^p < 0.05 or ^∗∗^*p* < 0.01 compared with the Control group.

Because inflammatory cytokines including IL-1β and IL-6 performed a vital role in the progression of hypertrophy, we assessed their expressions in heart to understand the effects of BAOXIN Granules. The results showed that the transcriptional levels of IL-1β and IL-6 significantly increased in myocardia after TAC surgery, and BAOXIN Granules or Enalapril treatment could significantly attenuate inflammatory response compared to mice treated with saline (Figures 4A,B). IL-1β protein level in the serum measured by ELISA also increased in TAC model and almost normalized by BAOXIN Granules or Enalapril treatment (Figure 4C).

### BAOXIN Granules Decreased Collagen Deposition in TAC Model

**FIGURE 5:**
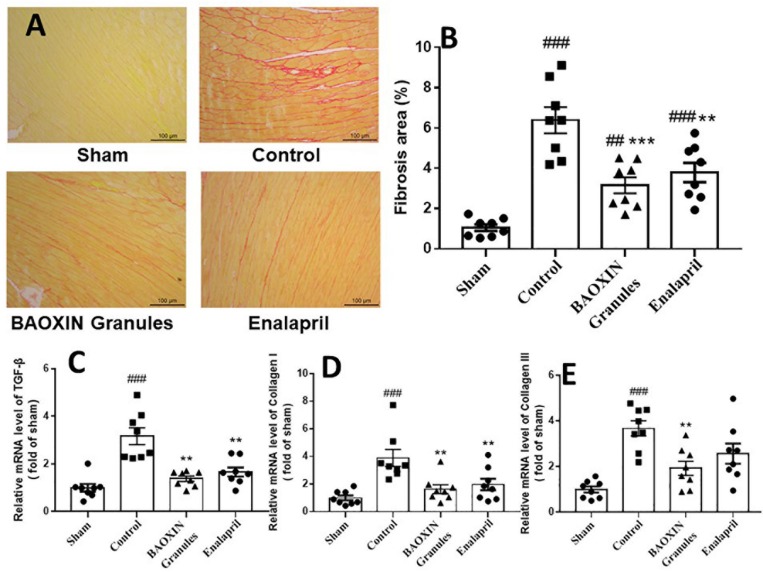
The effect of treatments on cardiac fibrosis in TAC-induced cardiac hypertrophy model. **(A)** Sirius Red staining of left ventricular tissue sections, 200X magnification. **(B)** Quantification of collagen in left ventricular by the Sirius Red staining sections. The mRNA expressions of the myocardial fibrosis related genes in heart tissue, TGF-β **(C)**, Collagen I **(D)** and Collagen III **(E)**. The numerical data were showed as mean and standard error of the mean (mean ± SEM). ${}^{\#\,\#}$*p* < 0.01 or ${}^{\#\,\#\,\#}$*p* < 0.001 compared with the Sham group. ^∗∗^*p* < 0.01 or ^∗∗∗^*p* < 0.001 compared with the Control group.

Sirius Red staining revealed collagen deposition in the saline-treated TAC mice (Figure 5A). BAOXIN Granules or Enalapril treatment groups had lesser cardiac fibrosis. Quantitative analysis showed that the fibrosis area of LV in BAOXIN Granules or Enalapril group was significantly lower than that in saline treated group after TAC induced (Figure 5B). Then quantitative real-time PCR analysis showed the expression of fibrosis related genes, including TGF-β, collagen I and collagen III, were strongly induced after TAC, but were inhibited after BAOXIN Granules and Enalapril treatment as Figure 5C.

## Discussion

Despite being beneficial at the initial stage to maintain the cardiac output, LV hypertrophy is progressively harmful, which leads to cardiomyopathy and heart failure (Vasan et al., 1997; Li et al., 2014). Therefore, it is of great importance to inhibit the progression of cardiac hypertrophy in an early stage. In recent years, more and more studies have demonstrated that TCM is protective in cardiovascular diseases (Lin et al., 2013; Liu et al., 2014; Wang et al., 2014a; Tao et al., 2015). BAOXIN Pills, which consist of multiple active components as such Salvianolic acid B, Tanshinone, and Astragaloside, has shown clinical benefits in improving cardiac function. However, one of the limitation of BAOXIN Pills is that it is simply prepared by pulverized crude drug and patients have to take a handful of Pills once to obtain effective dosage. BAOXIN Granules as a new preparation of BAOXIN Pills, has higher density of cardiac protective substances, less impurities, and more convenient administration.

In the present study, we used the preparation to intervene in the development of cardiac hypertrophy after TAC surgery in mouse as the model. TAC surgery is a widely used experimental method to induce pressure overload cardiac hypertrophy and heart failure, by binding the aortic arch between the innominate artery and the left carotid artery. Due to the chronic hemodynamic overload caused by TAC surgery, mice initially developed compensatory hypertrophy of the heart, and subsequently progressed to cardiac hypertroph and finally heart failure, if no effective interventions were taken (Koitabashi et al., 2011; Wei et al., 2012). This model mimics heart failure in humans, and helps to understand fundamental molecular processes and phenotypes critical in hypertrophy and heart failure (Weinberg et al., 1994; Yoo et al., 2018).

It was not surprising that BAOXIN Granules had ameliorated TAC-induced cardiac hypertrophy as significantly as Enalapril. Enalapril is a prodrug that can be hydrolyzed to release the active converting enzyme inhibitor enalaprilat by oral administration. As the positive control in this study, it was reported to be able to prevent cardiac hypertrophy in many animal models of heart failure (Knowles et al., 2001; Samuel et al., 2014). Our results also showed that BAOXIN Granules suppressed the overexpression of ANP, BNP, and β-MHC. Hence, we confirmed the cardio-protective effect of BAOXIN Granules in a mouse model of cardiac hypertrophy in early stage of heart failure.

Accumulated evidences have indicated that chronic inflammation is involved in the pathophysiology of hypertrophic remodeling (Frangogiannis, 2012; Wang et al., 2013; Yang et al., 2013; Chen et al., 2015). Pro-inflammatory cytokines, such as IL-1β (Toldo et al., 2013), IL-6 (Yu et al., 2005) and IL-13 (Wang et al., 2016), play a significant role in regulating the progression from adaptive hypertrophy to heart failure. Many effective constituents in BAOXIN Granules have been reported to inhibit the inflammatory responses. For example, Astragaloside could significantly inhibit inflammatory gene expression in LPS-treated mice (Zhang and Frei, 2015) to ameliorate acute pancreatitis (Qiu et al., 2015) and attenuate ovalbumin induced asthma (Huang et al., 2014b). Tanshinone (Li et al., 2015) and Angelica (Wang et al., 2015; Li et al., 2016) also exhibited anti-inflammatory effects by suppressing the secretion of pro-inflammatory cytokines. Therefore, it is easy to understand that BAOXIN Granules down-regulated inflammatory genes expressions, indicating that it protected against cardiac pathological changes through alleviating inflammatory response, though the underlying mechanisms remained to be clarified.

Increasing collagens and other extracellular matrix components can deposit in myocardium. Cardiac fibrosis is also an important hallmark of pathological hypertrophy and heart failure (Berk et al., 2007). The severity of interstitial fibrosis highly related to the extent of LV hypertrophy and impaired ejection fraction (Creemers and Pinto, 2011). In our study, histological examination demonstrated that LV collagen deposition was significantly reduced in two treated groups, and the decreased expression of TGF-β, Collagen I, and Collagen III also indicated anti-fibrotic effect of BAOXIN Granules. Some reports of the effective components in BAOXIN Granules showed the protective results for tissues fibrosis (Mao et al., 2014; Wang et al., 2014b; Li et al., 2017).

Various herbs in combination according to unique theory of TCM are considered that synergistic and complementary effects of different components on multiple targets supposedly have an advantage in clinical practice to enhance the curative efficacy and reduce the side effects. However, Chinese herbal compound is difficult to be analyzed chemically and by dose-effect relationship. It is also difficult to design the experiments to analyze the interaction of intricate network between complex components and human body. Hence, how to understand the mechanisms of TCM remains a problem to be resolved in the future. Our results will be helpful to explain the unique theory of TCM through the logic of the widely accepted.

## Conclusion

In summary, BAOXIN Granules could significantly inhibit the progression of cardiac hypertrophy and dysfunction induced by pressure overload in mice associated with inflammatory response and fibrosis. The results suggested BAOXIN Granules was expected to be developed to a new drug of TCM in China for heart failure treatment. Further studies are needed to explore additional mechanisms of BAOXIN Granules in apoptosis, energy metabolism, and so on. It is also unclear whether BAOXIN Granules has a role in preventing myocardial remodeling and followed cardiac dysfunctions in myocardial infarction models.

## Data Availability

The raw data supporting the conclusions of this manuscript will be made available by the authors, without undue reservation, to any qualified researcher.

## Ethics Statement

This study was carried out in accordance with the guide of Care and Use of Laboratory Animals published by the National Institutes of Health (NIH), United States. The protocol was approved by the Animal Care Committee of Peking University Health Science Center (No. LA2015012).

## Author Contributions

YW and GL conceived and designed the study. XQ and YW wrote the manuscript. XQ and JM performed most of the experiments and analyzed the data. JM and GJ analyzed the significance of the study in traditional Chinese medicine and clinical practice. ZD, XL, and HS participated in operation of the animal model and the ultrasonic examination. YS, DZ, and DL prepared the BAOXIN Granules and did the orthogonal test, and established the quality control standard.

## Conflict of Interest Statement

The authors declare that the research was conducted in the absence of any commercial or financial relationships that could be construed as a potential conflict of interest.
