# Supplementary material for: BAOXIN Granules Protected Mouse Model With Elevated Afterload From Cardiac Hypertrophy by Suppressing Both Inflammatory Reaction and Collagen Deposition
Source: Front Physiol. 2019 Jul 5;10:820. doi: 10.3389/fphys.2019.00820 (PMC6624790; doi:10.3389/fphys.2019.00820)
Supplement: Supplementary file 1 [file Data_Sheet_1.docx]

**Supplementary Materials**

***Preparation of BAOXIN Granules***

The herbal prescription of BAOXIN Granules contained 12 species of crude drugs and the percentage by weight of each drug listed as reported BAOXIN Pills previously (Pan Z et al., 1998) as following:

20% Astragalus membranaceus (Fisch.) Bunge, (Astragalus);

13% Salvia miltiorrhiza Bge, (Salvia miltiorrhiza);

10% Hiraute Shiny Bugleweed Herb, (Herba Lycopi);

10% Glycyrrhizae Radix et Rhizoma, (Glycyrrhiza);

10% Ophiopogon japonicus (Linn. f.) Ker-Gawl, (Ophiopogonis radix);

10% Poria cocos(Schw.) wolf, (Poria cocos);

7% Angelica sinensis (Oliv.) Diels, (Angelica);

7% Fructus Citri Aurantii Amarae L, (Fructus aurantii);

7% Rehmannia glutinosa (Gaetn.) Libosch. ex Fisch. et Mey., (Radix rehmanniae);

4% Platycodon grandiflorum(Jacq.), (Platycodon grandifloras);

4% Rheum palmatum L., (Cooked Rhubarb);

4% Whitm.ania Pigra Whitman, (Hirudo).

There are a lot of active compounds have been identified in 12 herbs. These compounds have been reported to affect many pathological progresses as list as the following table (supplementary Table 1). Therefore, although no pharmacological study for BAOXIN Pills has been carried out, the active ingredients may suggest that it is not surprise that its effect for cardiac hypertrophy in clinical report. However, the interactions of so many compounds have not been able to be analyzed yet, TCM had summarized a unique theory called *Compatibility of Medicines* for clinical practice.

BAOXIN Granules was prepared by Institute of Chinese Materia Medica, China Academy of Chinese Medical Sciences. Briefly, crude drugs of BAOXIN Granules were ground to superfine powder with via 80 mesh sieving for decoction, and extracted by ethanol solution. After filtering, the solution was concentrated and added excipients for vacuum drying and granulating. For extract process optimization, four factors at three levels were evaluated by orthogonal test as shown in supplemental table 2 and 3. The main active ingredients concentrations of BAOXIN Granules were tested by High-performance Liquid Chromatography (HPLC). Because 20% Astragalus and 13% Salvia miltiorrhiza are the main herbs in this prescription to play important roles as invigorating qi and activating *blood* according to the theory of TCM, the contents of Salvianolic acid B, Tanshinone IIA and Astragaloside in BAOXIN Granules were determined to calculate the transfer rate in the orthogonal test. The transfer rate (Y) was calculated as the formula Y= (R1/13S+R2/13T+R3/20A)×100%. R1, R2 and R3 are the weights (g) of Salvianolic acid B, Tanshinone IIA and Astragaloside extracted from 100g crude drug of herbal prescription respectively. S, T, A are the percentage contents (w/w) of Salvianolic acid B, Tanshinone IIA in the crude drug of Salvia miltiorrhiza and Astragaloside in the crude drug of Astragalus respectively. The levels of factors in Test 7 were used for preparation process of BAOXIN Granules.

***HPLC analysis***

To assess the quality stability of BAOXIN granules, the HPLC analysis and chromatographic separation was performed on an Agilent 1200 HPLC system (Agilent, USA) with Kromasil C18 column (4.6×250 mm, 5mm) (Akzo Nobel N.V, Sweden) at temperature of 35 °C. The mobile phase including methanol, [acetonitrile](http://dict.cn/acetonitrile), methanoic acid and H_2_O (25:10:1:64, v/v/v/v) with the flow rate of 1.0 ml/min was checked at 286 nm for UV signal. The standards were purchased from National Institute of Drug Control. The representative chromatogram is showed as the Supplementary Figure 1. We chose the Salvianolic acid B as the quality control for the preparation of BAOXIN Granules because of its highest concentration at 1.7% (w/w) by the decided process.

The preparation of BAOXIN Granules was controlled qualitatively with the content of Salvianolic acid B by HPLC assay (as shown as supplementary Figure 1) as the standard because its content is the highest.

***Monitoring body and heart weights***

The body weight was checked during the period of drugs administration for 4 weeks. There were no significant differences between the four groups during the experiments (as shown as supplementary Figure 2A). After TAC surgery, the heart weight (HW) to body weight (BW) ratio was significantly increased compared with the Sham group (as shown as supplementary Figure 2B) at the endpoint of the experiments 4 weeks later. It suggested the cardiac hypertrophy happened in the mice with increased afterload.

***Echocardiographic analysis***

Mice were anaesthetized through inhaling 1% isofluorane/oxygen for echocardiogram as described as the methods. Because we compared the cardiac function after treatment with the control treated with saline, we did not investigate the changes before and after surgery. Here, we showed the echocardiographic results of normal ICR mice and the ventricular wall thickness of the experimental groups (shown as Supplementary data table 5 and table 6).

Table 1. Main of the active compounds in BAOXIN Granules and their implications for cardiac hypertrophy

| Component | Main active compounds | Implications for hypertrophy treatment |
| --- | --- | --- |
| Astragalus | Astragaloside | Attenuate inflammatory cytokines (Komuro I., 2001)  Regulate energy biosynthesis (Zhang S et al., 2015) |
| Salvia miltiorrhiza | Tanshione | Prevented cardiac fibrosis (Maki T et al., 2002)  Modulate collagen metabolism (Ling S et al., 2009)  Reduce oxidative stress (Hu H et al., 2015) |
| Herba Lycopi | luteolin-7-O-beta-D- glucuronide methyl ester | Against pro-oxidant reactive species and low-density lipoprotein peroxidation (Lee MJ et al., 2010) |
| Glycyrrhiza | Glycyrrhizic acid and flavanones | Antioxidative activities (Carmeli and Fogelman, 2009)  Antimicrobial and Antiviral (Ryu et al.; Dao et al., 2010)  Anti-tumor (Ming LJ et al. 2013) |
| Ophiopogonis radix | Ophiopogonin D | Relieve mitochondrial damage (Zhang YY et al., 2015)  Activate potassium conductance (Ishibashi H et al., 2001) |
| Poria cocos | Sclederma | Duretic effect (Feng YL et al., 2013; Wu ZL et al., 2014) |
| Angelica | Angelan | Reverse Angiotensin II-induced apoptotic effects (Li X et al., 2014) |
| Fructusaurantii | Flavonoid glycosides | Anti-oxidant effect (Li X et al., 2015) |
| Radix rehmanniae | Catalpol | Attenuate oxidative/nitrative stress (Huang C et al., 2013) |
| Platycodon grandifloras | Triterpenoid saponins | Asthma, bronchitis, and pulmonary tuberculosis, and even as a sedative (Zhang et al., 2015)  Enhancing the exercise function (Kim et al., 2018)  Facilitating skeletal muscle protein synthesis and mitochondrial function ([Yong AnKim](https://www.sciencedirect.com/science/article/pii/S0278691518302850?via%3Dihub#!) et al., 2018) |
| Cooked Rhubarb | Chrysophanol、Rhein | Treatment of antiatherosclerosis(Chen J et al., 2009)  Improve diabetic conditions (Kasabri V et al., 2011) |
| Hirudo | Hirudin | Antimicrobial ([Kruer RM](https://www.ncbi.nlm.nih.gov/pubmed/?term=Kruer%20RM%5BAuthor%5D&cauthor=true&cauthor_uid=25423029) et al., 2015)  Anti-inflammatory ([AmirShakouri](https://www.sciencedirect.com/science/article/pii/S1744388117302542?via%3Dihub#!) et al., 2018)  Platelet inhibitory, anticoagulant and thrombin regulatory (Haycraft, et al., 1984; A. Michalsen et al., 2008) |

Table 2 Factors and levels

| Level | （A）  Extraction temperature (℃) | （B）  Extraction time (h) | (D)  Solid-liquid ratio | （C）  Frequency of extraction(n) |
| --- | --- | --- | --- | --- |
| 1 | 80 | 0.5 | 1/15 | 1 |
| 2 | 90 | 1 | 1/20 | 2 |
| 3 | 100 | 1.5 | 1/25 | 3 |

Table 3 Analysis of L_9_(3)^4^ test results of extract rates.

| Test No. | A | B | C | D | Y（%） |
| --- | --- | --- | --- | --- | --- |
| 1 | 1 | 1 | 1 | 1 | 16.44 |
| 2 | 1 | 2 | 2 | 2 | 23.03 |
| 3 | 1 | 3 | 3 | 3 | 22.51 |
| 4 | 2 | 1 | 2 | 3 | 24.41 |
| 5 | 2 | 2 | 3 | 1 | 31.39 |
| 6 | 2 | 3 | 1 | 2 | 25.43 |
| 7 | 3 | 1 | 3 | 2 | 34.59 |
| 8 | 3 | 2 | 1 | 3 | 30.47 |
| 9 | 3 | 3 | 2 | 1 | 24.51 |
| K1 | 61.98 | 75.44 | 72.34 | 72.33 |  |
| K2 | 81.23 | 84.88 | 71.94 | 83.05 |  |
| K3 | 89.56 | 72.45 | 88.48 | 77.38 |  |
| R | 27.58 | 12.43 | 16.54 | 10.72 |  |

Table 4 Primers for Real-Time Reverse Transcription–PCR

| Genes | Sense Primers | Antisense Primers |
| --- | --- | --- |
| ANP | GGGCTTCTTCTTCGTCTTGG | GGGGGCATGACCTCATCTTC |
| BNP | TAACGCACTGAAGTTGTTGTAGG | CGCTATGTTTATTATGTTGTGGC |
| β-MHC | CTTCACACCAGAAGAGAAGAACTC | CCCATGAGGTAGGCTGATTTG |
| IL-1β | AGGCTCCGAGATGAACAA | AAGGCATTAGAAACAGTCC |
| IL-6 | TTCTTGGGACTGATGCTG | CTGGCTTTGTCTTTCTTGTT |
| TGF-β | AGGAGACGGAATACAGGGCT | CCACGTAGTAGACGATGGGC |
| Collagen I | CCTGGCTCAAATGGCTCAC | CAGGACTGCCGTTATTCCCG |
| Collagen III | CCCTGGCTCAAATGGCTCA | TTCTTGCCACGCTTCCCTC |
| GAPDH | TGATGACATCAAGAAGGTGGTGAAG | TCCTTGGAGGCCATGTAGGCCAT |

Table 5 The echocardiographic analysis of the normal ICR mice (n=8)

| heart rate | EF(%) | FS(%) | LV Vol;s | LV Vol;d | LV Mass | LVAW;s (mm) | LVAW;d (mm) | LVPW;s (mm) | LVPW;d (mm) |
| --- | --- | --- | --- | --- | --- | --- | --- | --- | --- |
| 394.55±83.43 | 66.22±14.48 | 32.48±9.75 | 33.35±17.11 | 70.97±23.49 | 101.04±29.35 | 1.00±0.09 | 0.87±  0.12 | 0.93±  0.19 | 0.84±  0.07 |

The data were showed as average±SD.

Table 6 The heart rate and the heart wall thickness of each experimental group (n=8)

|  | Control | Baoxin Granules | Enalapril |
| --- | --- | --- | --- |
| heart rate | 463±70.65 | 499.6±74.70 | 547±49.67 |
| LVAW;s(mm) | 1.07±0.22 | 0.98±0.07 | 0.89±0.20 |
| LVAW;d(mm) | 0.90±0.04 | 0.87±0.17 | 0.77±0.22 |
| LVPW;s(mm) | 1.10±0.27 | 0.91±0.17 | 1.07±0.28 |
| LVPW;d(mm) | 0.99±0.17 | 0.91±0.24 | 0.91±0.31 |

The data were showed as average±SD.


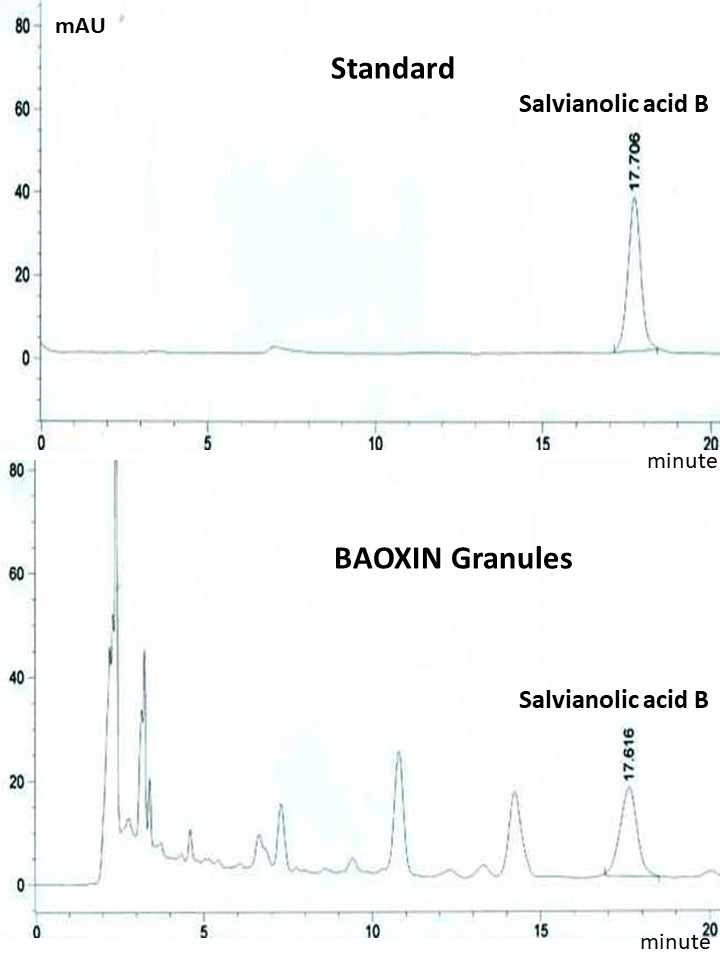


Figure 1. Above: HPLC chromatogram of Standard substance of Salvianolic acid B detected at 286 nm (tR 17.7 min). Below: Representative HPLC chromatogram of BAOXIN Granules preparation with Salvianolic acid B at an absorbance of 286 nm (tR 17.6 min).


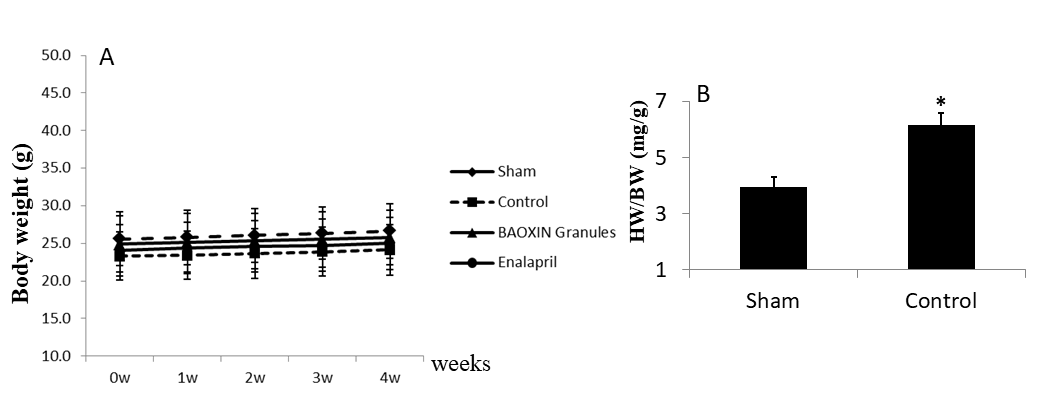


Figure 2. Monitoring body weights and heart weights. A. The changes of the body weight of four groups during the 4 weeks with the administration of drugs or saline. B. The ratio of hear weight (HW) to body weight (BW) showed that the heart became enlarged after the TAC surgery. * p<0.05
